# NSC114792, a novel small molecule identified through structure-based computational database screening, selectively inhibits JAK3

**DOI:** 10.1186/1476-4598-9-36

**Published:** 2010-02-11

**Authors:** Byung-Hak Kim, Jun-Goo Jee, Chang-Hong Yin, Claudio Sandoval, Somasundaram Jayabose, Daisuke Kitamura, Erika A Bach, Gyeong-Hun Baeg

**Affiliations:** 1 Department of Pediatrics, Division of Hematology/Oncology, New York Medical College, Valhalla, New York 10595, USA; 2 Center for Priority Areas, Tokyo Metropolitan University, 1-1 Minami-Osawa, Hachioji, Tokyo, Japan; 3 Division of Molecular Biology, Research Institute for Biological Sciences, Tokyo University of Science, Yamazaki 2669, Noda, Chiba, Japan; 4 Department of Pharmacology, New York University School of Medicine, New York 10016, New York, USA

## Abstract

**Background:**

Human or animals lacking either JAK3 or the common gamma chain (γc) expression display severe combined immunodeficiency disease, indicating the crucial role of JAK3 in T-cell development and the homeostasis of the immune system. JAK3 has also been suggested to contribute to the pathogenesis of tumorigenesis. Recent studies identified activating *JAK3 *mutations in patients with various hematopoietic malignancies, including acute megakaryoblastic leukemia. Importantly, functional analyses of some of those *JAK3 *mutations have been shown to cause lethal hematopoietic malignancies in animal models. These observations make JAK3 an ideal therapeutic target for the treatment of various human diseases. To identify novel small molecule inhibitors of JAK3, we performed structure-based virtual screen using the 3D structure of JAK3 kinase domain and the NCI diversity set of compounds.

**Results:**

We identified NSC114792 as a lead compound. This compound directly blocked the catalytic activity of JAK3 but not that of other JAK family members *in vitro*. In addition, treatment of 32D/IL-2Rβ cells with the compound led to a block in IL-2-dependent activation of JAK3/STAT5 but not IL-3-dependent activation of JAK2/STAT5. Consistent with the specificity of NSC114792 for JAK3, it selectively inhibited persistently-activated JAK3, but failed to affect the activity of other JAK family members and other oncogenic kinases in various cancer cell lines. Finally, we showed that NSC114792 decreases cell viability by inducing apoptosis through down-regulating anti-apoptotic gene expression only in cancer cells harboring persistently-active JAK3.

**Conclusions:**

NSC114792 is a lead compound that selectively inhibits JAK3 activity. Therefore, our study suggests that this small molecule inhibitor of JAK3 can be used as a starting point to develop a new class of drugs targeting JAK3 activity, and may have therapeutic potential in various diseases that are caused by aberrant JAK3 activity.

## Background

The mammalian genomes encode four members of the JAK family of protein tyrosine kinases, including JAK1, JAK2, JAK3, and TYK2 [1,2]. In particular, JAK3 is preferentially expressed in lymphoid cells and mediates signals through γc shared by receptors for IL-2, IL-4, IL-7, IL-9 and IL-15, indicating the crucial role of JAK3 in T-cell development and the homeostasis of the immune system [3]. Consistent with this observation, human or animals lacking either JAK3 or γc expression suffer from severe combined immunodeficiency disease characterized by the absence of T and NK cells and the presence of non-functional B cells [3]. Furthermore, JAK3 has been shown to be involved in the regulation of mast cell-mediated allergic and asthmatic responses [4]. Therefore, JAK3 has attracted significant attention in recent years as a therapeutic target for the treatment of various immune-related diseases such as autoimmune disorders and asthma, and for the prevention of organ allograft rejection [5,6].

In addition to the key role of JAK3 in immune cell development and function, it has also been suggested to contribute to the pathogenesis of tumorigenesis. Recent studies identified somatic mutations of *JAK3 *in a minority of acute megakaryoblastic leukemia patients [7-10], in a high-risk childhood acute lymphoblastic leukemia (ALL) case [11], and in cutaneous T-cell lymphoma patients [12]. Importantly, functional analyses of some of those *JAK3 *mutations have been shown to cause lethal hematopoietic malignancies in animal models [7], suggesting that those *JAK3 *mutations contribute to the pathogenesis of hematopoietic malignancies. In addition, persistently-activated JAK3 was reported in various cell lines that were derived from lymphoproliferative disorders, including mantle-cell lymphoma [13], Burkitt lymphoma [14], and anaplastic large-cell lymphoma [15-17]. Furthermore, it has been shown that persistently-activated JAK3 is observed in the mouse model of pre-B-cell leukemia spontaneously developed by loss-of-function of the tumor suppressor B-cell linker (BLNK) [18]. BLNK expression has been reported to be lost in 50% of pediatric B-ALL cases [19]. In addition, BLNK was shown to be required for direct JAK3 inhibition. These results suggest that persistent JAK3 activation contributes to the pathogenesis of a certain portion of pediatric B-ALL cases. Interestingly, despite the preferential expression of JAK3 in hematopoietic cells, persistently-activated JAK3 has also been reported in colon carcinoma tumors and cell lines [20], implying the role of JAK3 in the pathogenesis of solid tumors. In support of this, a recent study identified somatic *JAK3 *mutations in patients with breast carcinomas and gastric carcinoma [21]. Taken together, these findings make JAK3 an attractive therapeutic target for the treatment of patients with hematopoietic malignancies, as well as solid tumors.

In this study, we performed a small-scale, pilot structure-based computational database screen using the 3D structure of JAK3 kinase domain and the NCI diversity set of compounds to identify small molecule inhibitors of JAK3. We identified NSC114792 that potently inhibits both IL-2-induced and persistently-active JAK3. Importantly, this compound showed selective inhibition of JAK3 but not other JAK family members or other oncogenic kinases.

## Results

### Identification of NSC114792 through structure-based virtual screen

**Figure 1:**
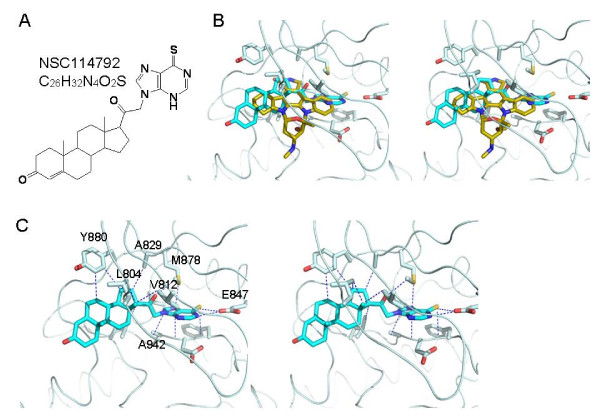
**Schematic diagrams of modeling of structure-based computational database screening**. (A) The chemical structure of NSC114792 (C_26_H_32_N_4_O_2_S; M.W., 464.6). Figures were generated by Pymol http://pymol.sourceforge.net. (B) Stereo structures of overlaid staurosporine analog AFN941 (4ST) and NSC114792 with JAK3 kinase domain (PDB ID: 1YVJ). 4ST is drawn in yellow and NSC114792 in cyan. (C) Predicted binding model of NSC114792 to the JAK3 kinase domain. Only the residues that contact NSC114792 are labeled. These contacts were selected only when two atoms in protein and ligand are located within 3.5Å.

To identify novel chemical compounds that inhibit JAK3 activity, we performed structure-based virtual screen using the 3D structure of JAK3 kinase domain (PDB ID: 1YVJ) and the NCI diversity set, which is a small library consisting of a collection of about 2,000 synthetic small molecules selected from the full NCI screening collection. We modified the conventional docking methods by generating several conformations of a compound and then utilizing the ensemble for docking. Our test runs revealed that the resulting complexes have the lower binding energies than those obtained by the simple increment of conformers. Of the compounds that showed lower binding energies in our virtual screening, we identified NSC114792 (10,13-dimethyl-17- [2-(6-sulfanylidene-3H-purin-9-yl)acetyl]-1,2,6,7,8,9,11,12,14,15,16,17 dodecahydrocyclopenta [a]phenanthren-3-one) (Figure 1A) as a potential JAK3 inhibitor due to its specificity for JAK3 over other JAK family members (see below). Its binding mode in the docked complex with JAK3 kinase domain is shown in Figure 1C. The lowest energy structure of NSC114792 displays the contacts in the side chains of Leu-804, Val-812, Ala-829, Lys-831, Glu-847, Val-860, Met-878, Tyr-880, Leu-932 and Ala-942 of the kinase domain, indicating that hydrophobic interaction is dominant. As shown in overlaid structures of 4ST and NSC114792 with JAK3 kinase domain (Figure 1B), the binding mode of NSC114792 to the JAK3 kinase domain is distinct from that of 4ST, where Val-812, Met-878, Tyr-880 and Leu-932 are considered the major contact sites. This observation suggests that additional residues around Tyr-880, Met-878 and Glu-847 in JAK3 kinase domain participate in binding of NSC114792. The values of dissociation constant, Kd, calculated by AutoDock energy were 10.64 and 5.44 nM for 4ST and NSC114792, respectively.

### NSC114792 directly blocks JAK3 kinase activity

**Figure 2:**
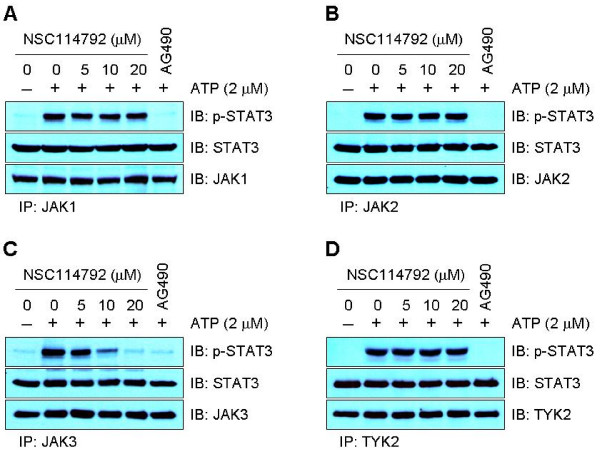
**NSC114792 selectively inhibits JAK3 kinase activity *in vitro***. JAK1 (A), JAK2 (B), JAK3 (C), or TYK2 (D) immunoprecipitates were used for *in vitro *kinase assay. Each immunoprecipitate was pre-incubated with either vehicle (DMSO) alone, NSC114792 at different concentrations or the pan-JAK kinase inhibitor AG490 (100 μmol/L) for 1 hour. Kinase reactions were subsequently performed by the addition of recombinant STAT3α protein as a substrate and 2 μmol/L ATP for 30 minutes at 30°C. The reaction products were processed for Western blotting and probed with antibodies specific for phospho-STAT3, STAT3, JAK1, JAK2, JAK3, and TYK2. Note that NSC114792 blocks JAK3 kinase activity in a dose-dependent manner but failed to inhibit the kinase activity of other JAK family members. STAT3, JAK1, JAK2, JAK3, and TYK2 serve as a loading control.

The four mammalian JAKs- JAK1, JAK2, JAK3, and TYK2 - share significant structural homology, which prompted us to investigate the specificity of NSC114792 for JAK3 and/or for other JAKs. We first performed *in vitro *kinase assays using immunoprecipitates for each JAK and recombinant STAT3α proteins as a substrate. JAK1, JAK2, and JAK3 immunoprecipitates were prepared from the lysates of Hodgkin's lymphoma HDLM-2 or L540 cells, where persistently-active JAK1 and JAK2 or JAK3 are expressed, respectively. Immunoprecipitates of TYK2 were derived from multiple myeloma U266 cells following treatment with IFN-α, a known activator of TYK2 [22]. Each immunoprecipitate was incubated with STAT3α protein in the absence or presence of various concentrations of NSC114792. All JAK immunoprecipitates were efficiently phosphorylated STAT3α protein in the absence of NSC114792. However, the addition of this compound resulted in an inhibition of JAK3 kinase activity in a dose-dependent manner (Figure 2C), whereas NSC114792 did not affect the kinase activity of other JAK members at the concentrations up to 20 μmol/L (Figure 2A, B and 2D). As expected, the pan JAK inhibitor AG490 blocked the kinase activity of all four JAKs.

**Figure 3:**
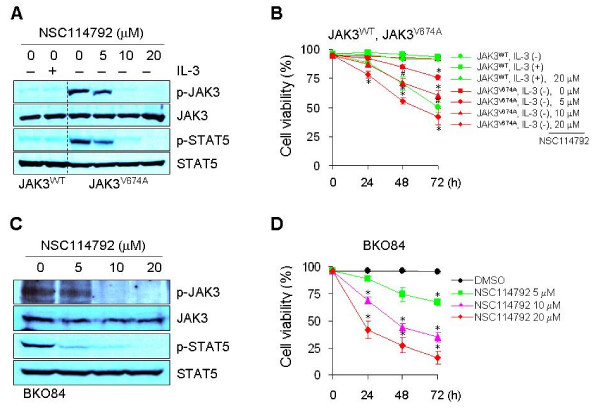
**NSC114792 affects the viability of BaF3 cells expressing an activating allele of *JAK3***. BaF3-JAK3^V674A ^cells were transformed by introducing an activating allele of *JAK3 *(V674A) [23]. (A) Treatment of BaF3-JAK3^V674A ^cells with NSC114792 results in a decrease in phosphorylated JAK3 and STAT5 levels in a dose-dependent manner. (B) NSC114792 decreases the viability of BaF3-JAK3^V674A ^cells in a time- and dose-dependent manner. BaF3-JAK3^WT ^cells were incubated for the indicated time periods in the absence or presence of IL-3, and with or without 20 μmol/L NSC114792. BaF3-JAK3^V674A ^cells were also cultured with NSC114792 at various concentrations for the indicated time periods. BaF3-JAK3^WT ^cells showed IL-3-dependent survival, and the viability of these cells was barely affected by 20 μmol/L NSC114792. By contrast, this dose of compound significantly decreased the viability of BaF3-JAK3^V674A ^cells. These data suggest that the effect of NSC114792 on BaF3-JAK3^V674A ^cell viability is not caused by the non-specific cytotoxicity of the compound. (C and D) NSC114792 blocks both JAK3 and STAT5 phosphorylation, and causes a significant decrease in cell viability in BLNK^-/- ^mice-derived BKO84 cells, which express constitutively-active JAK3/STAT5 due to the lack of BLNK inhibition of JAK3 [18]. (B) *, p < 0.001, indicates statistical significance compared to DMSO-treated BaF3-JAK3^V674 ^cells. #, p < 0.01, indicates statistical significance compared to IL-3-treated BaF3-JAK^WT ^cells. (D) *, p < 0.001, indicates statistical significance compared to DMSO-treated BKO84 cells.

A recent study identified an activating allele of *JAK3 *(V674A) from an acute myeloid leukemia patient-derived retroviral cDNA library, and showed that JAK3^V674A ^can transform the lymphoid pro-B-cell line BaF3 to IL-3-independent growth [23]. Since our compound showed ability to directly inhibit JAK3 kinase activity, treatment with the compound should block JAK3 activity in BaF3-JAK3^V674A ^cells. To test this hypothesis, we examined the effect of our compound on JAK3 phosphorylation in BaF3-JAK3^V674A ^cells. In BaF3-JAK3^WT ^cells, phospho-JAK3 was detected at a basal level and was not induced by IL-3 treatment (Figure 3A), consistent with the report that IL-3 regulates the proliferation and differentiation of hematopoietic cells through the tyrosine phosphorylation of JAK2 and not of JAK3 [24]. By contrast, in the absence of IL-3, persistently-active JAK3 was inhibited in a dose-dependent manner by treatment of BaF3-JAK3^V674A ^cells with NSC114792 (Figure 3A). In fact, a 10 μmol/L concentration of NSC114792 significantly abolished JAK3 phosphorylation. Since treatment with our compound led to a block in JAK3 phosphorylation in the cells, we expected to see a decrease in the levels of phosphorylated STAT5, which is a key downstream target of JAK3. Indeed, we found that the compound also inhibits phospho-STAT5 levels in a dose-dependent manner (Figure 3A). Since JAK3^V674A ^conferred IL-3-independent growth to BaF3-JAK3^V674A ^cells, we reasoned that the inhibition of this JAK3 should lead to a decrease in the viability of these cells. As predicted, treatment with NSC114792 decreased the viability of BaF3-JAK3^V674A ^cells in a time- and dose-dependent manner (Figure 3B). By contrast, BaF3-JAK3^WT ^cells showed near 100% viability in the presence of IL-3, and they were impervious to the effects of the compound, even at a 20 μmol/L concentration. These observations suggest that the decreased viability of BaF3-JAK3^V674A ^cells treated with NSC114792 was not caused by the non-specific cytotoxicity of this compound. We next determined that the IC_50 _value of NSC114792 in the growth of BaF3-JAK3^V674A ^cells is 20.9 μmol/L (Figure S1, Additional file 1). To verify that our compound's activities were not limited to BaF3 cells, we assessed its ability to inhibit JAK3 in pre-B leukemia cell line BKO84, which is derived from BLNK^-/- ^mice [18]. BLNK is a tumor suppressor that regulates IL-7-dependent survival of pre-B cells via direct inhibition of JAK3, indicating a crucial role of JAK3 in pre-B cell proliferation [18]. Consistent with this, treatment of BKO84 cells with anti-IL-7R-blocking antibody, which should decrease JAK3 activity, resulted in decreased cell viability [18]. To evaluate the effect of our compound on JAK3 activity in these cells, we cultured them with various concentrations of NSC114792. We found that treatment with NSC114792 decreased the tyrosine phosphorylation of both JAK3 and STAT5 in a dose-dependent manner (Figure 3C). Furthermore, we found that BKO84 cells treated with NSC114792 have significantly decreased viability in a time- and dose-dependent manner (Figure 3D). Taken together, our findings suggest that NSC114792 directly binds to JAK3 and inhibits its catalytic activity.

### NSC114792 blocks IL-2-induced JAK3/STAT5 signaling

JAK2 plays a pivotal role in signal transductions through the highly related receptors for cytokines and some hormones, including IL-3, prolactin (PRL), erythropoietin, granulocyte-macrophage colony-stimulating factor, and growth hormone [25,26]. By contrast, JAK3 is activated through the association with only the γc of IL-2, IL-4, IL-7, IL-9, IL-15 and IL-21 receptors [3,6,27]. To further evaluate the specificity of NSC114792 for JAK3 inhibition, we used the rat pre-T lymphoma cell line Nb2 and the murine myeloid progenitor cell line 32D stably expressing IL-2Rβ (32D/IL-2Rβ), both of which have been previously used to study cytokine-dependent activation of JAK proteins [28,29].

**Figure 4:**
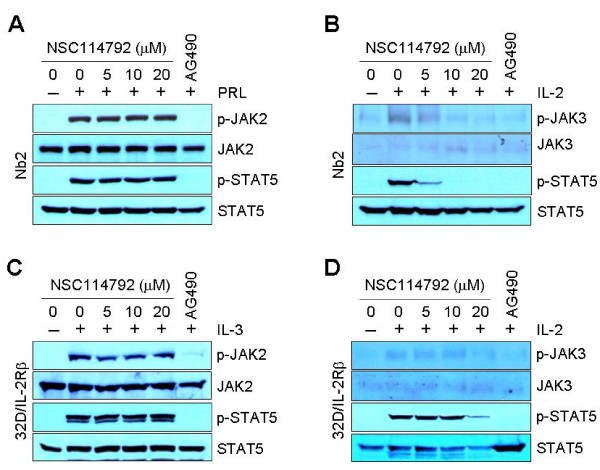
**NSC114792 inhibits IL-2-induced JAK3/STAT5 but not PRL- or IL-3-induced JAK2/STAT5 signaling**. Effects of NSC114792 on JAK2/STAT5 and JAK3/STAT5 signaling induced by PRL (prolactin) (A) and IL-2 (B), respectively, in the rat pre-T lymphoma cell line Nb2. Effects of NSC114792 on JAK2/STAT5 and JAK3/STAT5 signaling induced by IL-3 (C) and IL-2 (D), respectively, in the murine myeloid progenitor cell line 32D stably expressing IL-2Rβ (32D/IL-2Rβ). These cells were starved for 16 hours in the presence of either vehicle (DMSO) alone, NSC114792 at different concentrations, or the pan-JAK inhibitor AG490 (100 μmol/L). Nb2 cells and 32D/IL-2Rβ cells were subsequently stimulated by PRL (100 ng/mL) or IL-2 (100 ng/mL) for 10 minutes, and IL-3 (5 ng/mL) or IL-2 (100 ng/mL) for 30 minutes, respectively. In Nb2 cells, NSC114792 decreased IL-2-induced phospho-JAK3 and -STAT5 levels in a dose-dependent manner, but failed to decrease PRL-induced phospho-JAK2 and -STAT5 levels. Similarly, in 32D/IL-2Rβ cells, this compound blocked IL-2-induced phospho-JAK3 and -STAT5, but was defective in inhibiting IL-3 induced phospho-JAK2 and -STAT5. AG490, a pan-JAK inhibitor, non-selectively blocked both phospho-JAK2 and -JAK3 in all cells.

We first examined the effects of NSC114792 on phospho-JAK2 and phospho-JAK3 induced by PRL and IL-2 treatment, respectively, in Nb2 cells. Cells were incubated in the presence of NSC114792 for 16 hours and then stimulated by PRL or IL-2 for 10 minutes. While phospho-JAK2 and phospho-JAK3 were barely detectable in cells without stimulation, their levels were increased in response to PRL and IL-2 stimulation, respectively (Figure 4A and 4B). As expected, NSC114792 could not inhibit PRL-induced JAK2/STAT5 phosphorylation at the concentrations up to 20 μmol/L (Fig. 4A). By contrast, it did block IL-2-induced JAK3/STAT5 phosphorylation in a dose-dependent manner (Figure 4B). In fact, IL-2-induced phospho-STAT5 levels were decreased by more than 80% at a 5 μmol/L of NSC114792 compared with those of control, and undetectable at a 10 μmol/L (Figure 4B). By contrast, treatment of Nb2 cells with AG490 resulted in a profound reduction of both PRL-induced JAK2/STAT5 and IL-2-induced JAK3/STAT5 phosphorylation, due to its ability to inhibit all JAKs. The selective effect of NSC114792 on JAK3/STAT5 signaling in Nb2 cells was further demonstrated in 32D/IL-2Rβ cells. In these cells, JAK2 and JAK3 are activated by IL-3 and IL-2 treatment, respectively [29]. Cells were treated with NSC114792 for 16 hours and then stimulated with IL-3 or IL-2 for 30 minutes. In 32D/IL-2Rβ cells in the absence of cytokine stimulation, phospho-JAK2 and phospho-JAK3 were barely detectable. However, consistent with the previous report, JAK2 and JAK3 become tyrosine phosphorylated in response to treatment with IL-3 and IL-2, respectively (Figures 4C and 4D). Consistent with the results from Nb2 cells, NSC114792 did not affect IL-3-induced JAK2/STAT5 phosphorylation (Figure 4C), whereas it did block IL-2-induced JAK3/STAT5 phosphorylation (Figure 4D). Once again, the pan-JAK inhibitor AG490 non-selectively inhibited JAK2 and JAK3 phosphorylation induced by IL-3 and IL-2, respectively. These findings strongly suggest that NSC114792 has selectivity for JAK3 over JAK2.

### NSC114792 inhibits persistently-active JAK3

**Figure 5:**
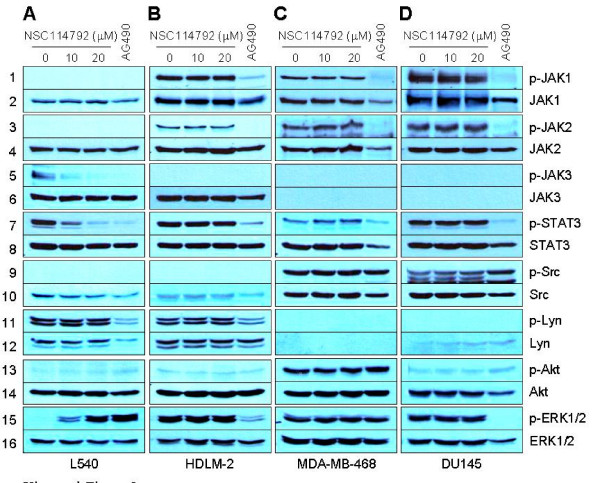
**NSC114792 selectively inhibits persistently-active JAK3/STAT signaling in cancer cells**. The Hodgkin's lymphoma cell lines L540 (A) and HDLM-2 (B), the breast cancer cell line MDA-MB-468 (C), and the prostate cancer cell line DU145 (D) were cultured for 24 hours in the presence of either vehicle (DMSO) alone, NSC114792 at various concentrations up to 20 μmol/L, or the pan-JAK inhibitor AG490 (150 μmol/L). Whole-cell extracts were processed for Western blot analysis using antibodies specific for the molecules indicated. Treatment with NSC114792 did not alter the levels of phospho-JAK1 and -JAK2 (B-D, lanes 1-4), but it did potently inhibit phospho-JAK3 in a dose-dependent manner (A, lanes 5 and 6). Consistently, the compound decreased phospho-STAT3 only in L540 cells, which exhibit persistent JAK3 activation (lanes 7 and 8). In contrast, AG490 non-selectively decreased the phosphorylation of JAK kinases and thus led to a block in phospho-STAT3 in all cell lines tested. NSC114792 did not alter the levels of other activated oncogenic kinases, including phospho-Src (lanes 9 and 10), phospho-Lyn (lanes 11 and 12), phospho-Akt (lanes 13 and14), and phospho-ERK1/2 (lanes 15 and 16). None of these kinases were affected by treatment with NSC114792 at concentrations up to 20 μmol/L.

We further assessed if NSC114792 can specifically inhibit JAK3, but not other JAKs, using various cancer cell lines where constitutively-active JAK kinases are expressed. Hodgkin's lymphoma L540 cells had high levels of phospho-JAK3 but undetectable levels of phospho-JAK1 and -JAK2 (Figure 5A). In contrast, Hodgkin's lymphoma HLDM-2 cells, breast cancer MDA-MB-468 cells and prostate cancer DU145 cells exhibited high levels of phospho-JAK1 and -JAK2 but not phospho-JAK3 (Figure 5B-D). We assessed if NSC114792 can inhibit the persistently-active JAK kinases in these cells. Treatment of L540 cells with NSC114792 caused a reduction of phospho-JAK3 levels in a dose-dependent manner, whereas this compound did not alter the total JAK3 levels (Figure 5A, lanes 5 and 6). We found that L540 cells treated with 10 μmol/L NSC114792 exhibited more than a 70% decrease in the phospho-JAK3 levels, compared with those of control. Moreover, when L540 cells were treated with 20 μmol/L NSC114792, JAK3 phosphorylation was almost completely abolished. By contrast, the compound did not alter phospho-JAK1 and -JAK2 levels in HDLM-2, MDA-MB-468, and DU145 cells (Figure 5B-D, lanes 1-4). In addition, NSC114792 did not inhibit IFN-α-induced TYK2 phosphorylation in U266 cells at the concentrations up to 20 μmol/L (Figure S2, Additional file 2). As expected, AG490 profoundly reduced the phosphorylation levels of all JAKs tested in those cells (Figure 5A-D, lanes 1-6; Figure S2, Additional file 2). Our results thus far indicate that NSC114792 selectively inhibits JAK3. To assess the functional outcome of this inhibition, we monitored the phosphorylation of a JAK3 target. We chose STAT3, which is phosphorylated by JAKs on Y705, as its persistent activation is the most common STAT form found in human cancers [30]. We found that NSC114792 inhibits phospho-STAT3 levels in a dose-dependent manner in L540 cells, which have elevated phospho-JAK3 levels (Figure 5A, lanes 7 and 8). In contrast, at the concentrations up to 20 μmol/L, NSC114792 did not inhibit the phosphorylation of STAT3 in cells that lack persistently-active JAK3 (i.e., HDLM-2, MDA-MB-468, and DU145 cells) (Figure 5B-D, lanes 7 and 8). As predicted, treatment of all cell lines with AG490 resulted in a dramatic decrease in phospho-STAT3 levels in all cell lines tested (Figure 5A-D, lanes 7 and 8).

Members of the Src family of non-receptor tyrosine kinases can activate STAT3 by phosphorylating Y705 [31]. To assess if our compound can inhibit Src family kinases, we monitored the tyrosine phosphorylation state of Src and Lyn. NSC114792 did not reduce the levels of phospho-Lyn in L540 and HDLM-2 cells or the levels of phospho-Src in MDA-MB-468 and DU145 cells at any concentration tested (Figure 5A-D, lanes 9-12). We further examined whether NSC114792 can affect other oncogenic signaling pathway components, such as the serine/threonine kinase Akt or MAPK (also called ERK1/2). We detected no significant inhibitory effects of our compound on phospho-Akt and phospho-ERK1/2 levels in all cell lines tested (Figure 5A-D, lanes 13-16). Taken together, our results indicate that NSC114792 selectively inhibits JAK3 activity and subsequently leads to a block in STAT signaling.

### NSC114792 selectively inhibits the viability of cancer cells with constitutively-active JAK3

**Figure 6:**
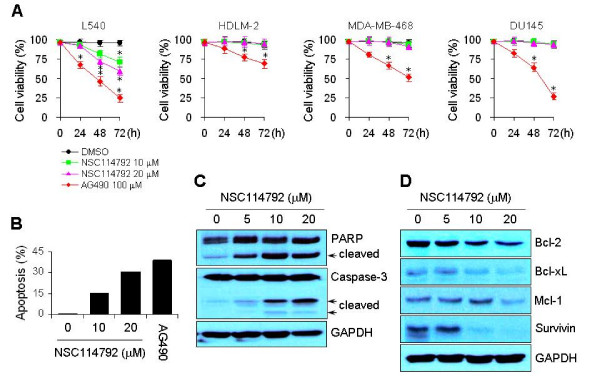
**NSC114792 decreases cell viability and induces apoptosis in cancer cells with persistent JAK3 activation**. (A) NSC114792 decreases cell viability only in L540 cells that express persistently-active JAK3. L540, HLDM-2, MDA-MB-468 and DU145 cells were treated with either vehicle (DMSO) alone, NSC114792 at different concentrations or the pan-JAK inhibitor AG490, and incubated for the indicated time periods. The trypan blue exclusion assay was performed to count viable cells. Results are shown as the mean of three independent experiments (± SD indicated by error bar). *, p < 0.001, indicates statistical significance compared to DMSO-treated cells. (B) TUNEL assay shows that NSC114792 induces apoptosis in L540 cells. Cells were treated with vehicle (DMSO) alone or NSC114792 at various concentrations for 72 hours. Cells were then stained with FITC-conjugated BrdU antibody and propidium iodide (PI), and subsequently subjected to flow cytometry. The percentages of TUNEL-positive cells are indicated. (C and D) Treatment with NSC114792 increases cleaved fragments of Poly (ADP-ribose) polymerase (PARP) and caspase-3, and decreases the expression of anti-apoptotic genes in a dose-dependent manner in L540 cells. Cells were treated with vehicle (DMSO) alone or NSC114792 at various concentrations for 48 hours. Whole-cell extracts were processed for Western blot analysis using antibodies specific for the molecules indicated. GAPDH serves as a loading control.

Small molecule inhibitors of JAK/STAT signaling have been shown to repress cell proliferation by affecting cell viability in a variety of solid tumor cell lines, as well as in blood malignant cell lines, suggesting the critical role of JAK/STAT signaling in the proliferation of cancer cells [32-35]. Because NSC114792 selectively inhibited JAK3/STAT signaling, we hypothesized that treatment with our compound would affect cell viability only in cancer cells that express constitutively-active JAK3/STATs. We assessed if NSC114792 can reduce viability of L540, HDLM-2, MDA-MB-468, and DU145 cells. Cells were treated with either vehicle (DMSO) alone, NSC114792 at different concentrations or AG490, and they were incubated for various time periods. We found that NSC114792 decreases cell viability only in L540 cells with persistent JAK3 activation, in a time- and dose-dependent manner, but not in HDLM-2, MDA-MB-468 and DU145 which lack persistently-active JAK3 (Figures 5 and 6A). In contrast, treatment with the pan-JAK inhibitor AG490 significantly reduced cell viability in all cell lines tested (Figure 6A).

### NSC114792 induces apoptosis via down-regulating the expression of anti-apoptotic genes

We previously reported that treatment L540 cells with siRNA against *JAK3 *causes an increase in the cleavage of PARP and caspase-3, and a decrease in the expression of anti-apoptotic genes [36], suggesting that knockdown of JAK3 activity closely correlates with apoptosis in L540 cells. To demonstrate that NSC114792 affected cell viability by inducing apoptosis, we performed TUNEL assay on L540 cells. We found that treatment with NSC114792 induces apoptosis in a dose-dependent manner in L540 cells and that the number of TUNEL-positive cells increased more than 30-fold in cells treated with 20 μmol/L NSC114792 compared with controls (Figure 6B). To gain more insights into the molecular mechanism by which NSC114792 induces apoptosis in L540 cells, we assessed if it can induce an increase in the cleavage of PARP and caspase-3, both of which are hallmarks of apoptosis [37]. As expected, treatment with the compound increased both PARP and caspase-3 cleaved fragments in a dose-dependent manner (Figure 6C). We next examined the effect of this compound on the expression of anti-apoptotic genes, which are known STAT targets. L540 cells were treated with NSC114792 for 48 hours, and then the whole-cell extracts were processed for Western blot analysis using antibodies specific for Bcl-2, Bcl-xL, Mcl-1, and Survivin. The expression of these proteins was inhibited by treatment with NSC114792 in a dose-dependent manner, whereas the levels of GAPDH remained unchanged (Figure 6D). These results indicate that in L540 cells NSC114792 inhibits JAK3/STAT signaling and thus decreases cell survival by inducing apoptosis through down-regulating the expression of anti-apoptotic genes.

## Discussion

In this study, we performed a small-scale, pilot structure-based computational database screen using the molecular docking program AutoDock (Version 4.0) for compounds that dock into the catalytic site of JAK3 kinase domain. This screening resulted in the identification of NSC114792 as a lead compound that specifically inhibits the catalytic activity of JAK3 but not that of other JAK family members. Our results indicate that the mechanism by which NSC114792 inhibits JAK3 involves direct interaction between this small molecule and the JAK3 kinase domain. *In vitro *kinase assays revealed that addition of this compound to the JAK3 immunoprecipitates causes a significant block in JAK3 kinase activity. Furthermore, the inhibition of JAK3 by this compound was disrupted in the presence of excess ATP, indicating that NSC114792 is an APT-competitive JAK3 inhibitor (data not shown). Notably, this compound was defective in inhibiting the kinase activity of other JAKs, even at a concentration that almost completely abolished JAK3 kinase activity. The specificity of NSC114792 for JAK3 over other JAK kinases was further supported by our docking simulation. Of the homologous sequences that were retrieved by BLAST search based on the sequence of JAK3 kinase domain (1YVG), we identified five with reported structures. The PDB codes of these are: 3EYG and 3EYH for JAK1 kinase, and 2B7A, 3E62 and 3FUP for JAK2 kinase. We attempted the docking simulation of NSC114792 toward these structures. We found the value of dissociation constant, Kd, calculated by AutoDock energy for 1YVG/NSC114792 (i.e., JAK3) was 5.44 nM. By contrast, the dissociation constants were: 40.25 nM (3EYG/NSC114792) and 18.68 nM (3EYH/NSC114792) for JAK1; and 17.47 nM (2B7A/NSC114792), 18.82 nM (3E62/NSC114792), and 36.95 nM (3FUP/NSC114792) for JAK2. These observations suggest that the binding affinity of NSC114792 to the JAK3 kinase domain is at least 3-fold higher to those of JAK1 and JAK2. We next performed a detailed analysis to seek for possible reasons for the high selectivity of NSC114792 for JAK3 over other JAK kinases. We compared the ligand-binding pockets in all JAK proteins and superimposed the ligand structures onto the pockets. Our analysis showed that the purine moiety of NSC11492 fits snugly into a cleft comprised of Ala-829, Lys-831, Glu-847, Val-860, Met-878, Ala-942, Asp-943 and Phe-944 in JAK3 kinase domain. While most of these residues are conserved in JAK1, JAK2 and JAK3, Ala-942 is unique to JAK3. In JAK1 and JAK2, a Gly residue is found in the analogous position of Ala-942. We found that the methyl group of Ala-942 forms hydrophobic contacts with the purine moiety of NSC114792. To examine the role of the methyl group on Ala-942-NSC114792 interactions, we performed *in silico *docking experiments on a JAK3 kinase domain in which Ala-942 was mutated to Gly. Interestingly, the calculated binding free energy between NSC114792 and JAK3 kinase domain dropped from 5.44 nM to 74.16 nM. This observation suggests that Ala-942 in the JAK3 kinase domain is the key residue determining the specificity of NSC114792 for JAK3. To demonstrate the selectivity of NSC114792 for JAK3, we also showed that NSC114792 inhibits the tyrosine phosphorylation of JAK3 and decreases cell viability only in cancer cells harboring persistently-activated JAK3. The reduced cell viability is likely due to a decrease in the expression of anti-apoptotic genes because treatment of L540 cells with NSC114792 resulted in a significant increase in the apoptosis and a concomitant decrease in the expression of Bcl-2, Bcl-xL and other factors that block programmed cell death. By contrast, this compound had no effect on cancer cells that lack persistently-activated JAK3. Interestingly, our compound did not alter the levels of phosphorylated forms of other oncogenic kinases, such as Src, Akt and ERK1/2. Although the specificity of NSC114792 for JAK3 over other oncogenic kinases still needs to be fully examined by evaluating its effects on a large panel of tyrosine and serine/threonine kinases *in vitro*, our findings strongly suggest that it selectively inhibits JAK3.

Recent studies identified somatic mutations of *JAK3 *in a minority of acute megakaryoblastic leukemia patients [7-10], in a high-risk childhood acute lymphoblastic leukemia case [11], and in cutaneous T-cell lymphoma patients [12]. Importantly, functional analyses of many of those identified *JAK3 *mutations showed that each of the mutations can transform BaF3 cells to factor-independent growth and can cause lethal hematopoietic malignancies in murine bone marrow transplantation models [7], suggesting that somatic *JAK3 *mutations contribute to the pathogenesis of various hematopoietic malignancies. These findings strongly demonstrate that JAK3 can serve as a logical target for therapeutic intervention in the hematopoietic malignancies with activating alleles of *JAK3*. In contrast to the role of gain-of-function of *JAK3 *in the pathogenesis of hematopoietic malignancies, *JAK3 *deficiency in mice and human causes immunodeficiency, indicating the pivotal role of JAK3 in the immune system [6,38,39]. In fact, recently developed JAK3 inhibitors, including CP-690550, PNU156804 and R348, can function as immunosuppressive agents [40-42]. These compounds have been shown to inhibit cytokine-induced JAK3 activity and significantly prolong survival in animal models for organ transplantations. Taken together, small molecule inhibitors that can selectively block JAK3 activity may have enormous therapeutic value in several immune-related diseases including organ allograft rejection, as well as in lymphoproliferative disorders with aberrant JAK3 activation.

## Conclusions

As the protein structure determination methodology advances, the use of a structure-based drug discovery approach is becoming more popular due to the possibility to screen millions of molecules in a timely way [43]. NSC114792, a novel small molecule identified through structure-based computational database screen, potently inhibits both cytokine-induced and constitutively-active JAK3. Importantly, this compound exhibited selectivity for JAK3 over other JAK family members and other oncogenic signaling pathway components. These results indicate the robustness and validity of our structure-based virtual screen.

Finally, our study strongly suggests that NSC114792 or its derivatives can be used as a lead compound to develop new group of drugs targeting JAK3, and may have therapeutic potential in human immune-related diseases and hematopoietic malignancies that are caused by aberrant JAK3 activity.

## Methods

### Structure-based virtual screen

To discover compounds that inhibit JAK3 activity, we employed AutoDock version 4 [44] and performed virtual screening with the NCI diversity set of compounds. The protein coordinate from the complex structure (PDB ID: 1YVJ) between the JAK3 kinase domain and its inhibitor staurosporine analog AFN941 (4ST) [45] was chosen for virtual screening. After removing the ligand and solvent molecules from the complex structure, hydrogen atoms were added. Ionizable states in Asp, Glu, His, and Lys residues were considered by PDB 2PQR[46]. The docking simulation of a compound starts with defining 3D potential grids for the receptor protein against the atom types of a compound. The calculated grid maps were of dimension 40 × 40 × 40 points with the spacing of 0.375 Å. For the parameters of generic algorithm in AutoDock version 4, we used 100 and 500,000 for the number of individuals in population (*ga_pop_size*) and the maximum number of generations (*ga_num_evals*), respectively. A docking for each compound was repeated 10 times with different initial conformations that were generated by AMBER [47], and the conformations and energies in the 10 runs were clustered together. All the procedures in the virtual screening were carried out in automatic way using in-house written scripts. As proof of principle, we assessed if 4ST, a known substrate of JAK3, could bind to the kinase domain using our method. The docked conformation of 4ST was in excellent agreement with the bound conformation in the crystal structure, showing the pairwise root mean square deviation value of 0.70 Å. Once completing virtual screen, the final results were ranked on the bases of the predicted binding free energy and the cluster size for each docking conformation.

### NSC114792

NSC114792 is one of the compounds identified from the NCI diversity set of compounds, which have been deposited to the Developmental Therapeutics Program (DTP)/NCI by the outside originators of the materials and have been available to investigators for non-clinical research purposes. The information on the synthesis of NSC114792 and its purity is not available from the DTP/NCI website at the time of re-submission.

### Cell lines and culture conditions

The Hodgkin's lymphoma cell lines L540 and HLDM-2 were obtained from the German Collection of Microorganisms and Cell Cultures (DSMZ, Germany) and maintained in RPMI 1640 containing 20% FBS. The breast cancer cell line MDA-MB-468, the prostate cancer cell line DU145 and the multiple myeloma cell line U266 were purchased from the American Type Culture Collection (Rockville, MD). MDA-MB-468 and DU145 cells were maintained in DMEM containing 10% FBS, and U266 cells were maintained in RMPI1640 containing 10% FBS. Bone marrow-derived pro-B-cell line BaF3 stably expressing wild type *JAK3 *or mutant *JAK3 *(V674A) were obtained from Dr. Hiroyuki Mano [23] and maintained in RPMI 1640 containing 10% FBS. Pre-T lymphoma Nb2 cells were obtained from Dr. Charles V. Clevenger [48], and cultured in RPMI 1640 containing 10% FBS and 5 mM HEPES buffer, pH 7.3. Myeloid progenitor 32D cells stably expressing IL-2Rβ were obtained from Drs. Achsah D. Keegan and Warren J. Leonard [49,50], and maintained in RPMI 1640 medium containing 10% FBS and 5% WEHI-3B cell-conditioned medium as a source of IL-3. BKO84 cells were cultured in RPMI1640 containing 10% FBS, 55 μM 2-ME, and 500 μg/mL G418 [18]. All the cells were cultured at 37°C in a humidified incubator containing 5% CO_2_.

### Western blot analysis and antibodies

Cell pellets were lysed in a lysis buffer [50 mM Tris-HCl, pH 7.4, 350 mM NaCl, 1% Triton X-100, 0.5% Nonidet P-40, 10% glycerol, 0.1% SDS, 1 mM EDTA, 1 mM EGTA, 1 mM Na_3_VO_4_, 1 mM phenylmethylsulphonyl fluoride, and phosphatase inhibitor cocktail]. Whole-cell extracts were resolved on SDS-PAGE, transferred to nitrocellulose membrane, and probed with appropriate antibodies. Antibodies specific for phospho-JAK3, JAK3, STAT3, STAT5 and Lyn were purchased from Santa Cruz Biotechnology (Santa Cruz, CA). Antibodies specific for phospho-STAT3, phospho-STAT5, JAK1, phospho-JAK2, JAK2, phospho-TYK2, TYK2, phospho-Src, Src, phospho-Lyn, phospho-Akt, Akt, phospho-ERK1/2, ERK1/2, PARP, caspase-3, Bcl-2, Bcl-xL, Mcl-1, Survivin and GAPDH were purchased from Cell Signaling Technology (Cambridge, MA). Phospho-JAK1 antibody was obtained from Upstate Chemicon (Temecula, CA). Membranes were blocked in 5% non-fat dried milk in Tris-buffered saline containing 0.1% Tween 20 for 1 hour and subsequently incubated with primary antibodies at 4°C for overnight. Membranes were then probed with horseradish peroxidase-conjugated secondary antibodies (GE Healthcare, Piscataway, NJ), and then visualized by Enhanced Chemiluminescence Reagent (GE Healthcare, Piscataway, NJ).

### Cell viability and apoptosis assay

Cell viability was determined by the trypan blue exclusion assay. Briefly, cells (5 × 10^4 ^cells/mL) were treated with either vehicle (DMSO) alone, NSC114792 at different concentrations or AG490 (100 μmol/L), and incubated for the indicated time periods. For performing apoptosis assay, TUNEL assay was conducted as previously described [36]. Briefly, L540 cells (1.0 × 10^6 ^cells/mL) were treated with either vehicle (DMSO) alone or NSC114792 (10 or 20 μmol/L) for 72 hours, stained using an APO-BRDU™ kit, according to the manufacture's protocol (Phoenix Flow Systems, San Diego, CA), and then subsequently subjected to Elite ESP flow cytometry (Coulter Inc., Miami, FL).

### In vitro kinase assay

Recombinant His-tagged STAT3α protein was purified as previously described [36] and used as a substrate for *in vitro *kinase assays. For *in vitro *JAK kinase assays, L540, HDLM-2 and IFN-α-stimulated U266 cells were lysed in a lysis buffer [20 mM Tris-HCl (pH 7.4), 500 mM NaCl, 0.25% Triton X-100, 1 mM EDTA, 1 mM EGTA, 10 mM β-glycerophosphate, 1 mM DTT, 300 μM Na_3_VO_4_, 1 mM phenylmethylsulphonyl fluoride, and phosphatase inhibitor cocktails] on ice. The lysates were pre-cleared with protein A/G-sepharose for 2 hours at 4°C and then incubated with anti-JAK1, anti-JAK2, anti-JAK3 or TYK2 antibodies for overnight at 4°C. The immune complexes were subsequently precipitated by protein A/G-sepharose beads. The precipitates were washed twice with kinase buffer [25 mM Tris/HCl (pH 7.5), 20 mM β-glycerophosphate, 10 mM MgCl_2_, 2 mM DTT, 1 mM Na_3_VO_4_, and protease inhibitor cocktail]. The immune complexes were mixed with either vehicle (DMSO) alone, NSC114792 at different concentrations or the pan-JAK inhibitor AG490 for 1 hour at 30°C. Kinase reactions were subsequently performed by the addition of 2 μg His-tagged STAT3α proteins in the absence or presence of ATP (2 μmol/L) for 30 minutes at 30°C. The reaction products were subjected to SDS-PAGE and probed with antibodies specific for phospho-STAT3, STAT3, JAK1, JAK2, JAK3, or TYK2.

## Competing interests

The authors declare that they have no competing interests.

## Authors' contributions

BK, JJ, and CY performed the experiments. GB conceived the experimental design and wrote the paper. CS, SJ, and EB critically revised the paper. DK provided the reagents. All authors read and approved the final manuscript.

## Supplementary Material

Additional file 1 **Figure S1 - NSC114792 inhibits cell growth in BaF3-JAK3^V674A ^cells**. BaF3-JAK3^V674A ^cells were treated with either vehicle (DMSO) alone or NSC114792 at various concentrations up to 40 μmol/L, and incubated for 48 hours. Note that the IC_50 _value of NSC114792 is 20.9 μmol/L. Results are shown as the mean of three independent experiments (± SD indicated by error bar). *, p < 0.001. Click here for file

Additional file 2 **Figure S2 - Treatment with NSC114792 has no effect on TYK2 phosphorylation**. U266 cells were cultured for 24 hours in the presence of either vehicle (DMSO) alone, NSC114792 at different concentrations or the pan-JAK inhibitor AG490 (150 μmol/L), and then stimulated with 1000 U/mL IFN-α for 30 minutes. Whole-cell extracts were processed for Western blot analysis using phospho-TYK2 and TYK2 antibodies. GAPDH serves as a loading control. Click here for file
