# Supplementary figures and images for: NSC114792, a novel small molecule identified through structure-based computational database screening, selectively inhibits JAK3
Source: Mol Cancer. 2010 Feb 11;9:36. doi: 10.1186/1476-4598-9-36 (PMC2830973; doi:10.1186/1476-4598-9-36)

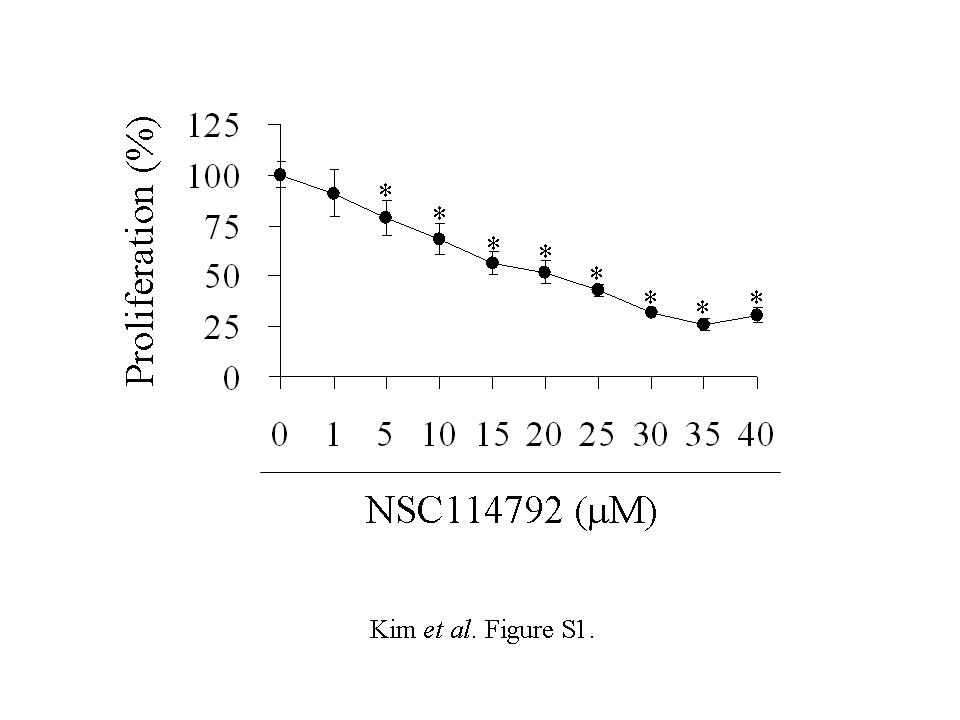

Supplement: Additional file 1 — Figure S1 - NSC114792 inhibits cell growth in BaF3-JAK3V674A cells. BaF3-JAK3V674A cells were treated with either vehicle (DMSO) alone or NSC114792 at various concentrations up to 40 μmol/L, and incubated for 48 hours. Note that the IC50 value of NSC114792 is 20.9 μmol/L. Results are shown as the mean of three independent experiments (± SD indicated by error bar). *, p < 0.001. [file 1476-4598-9-36-S1.JPEG]

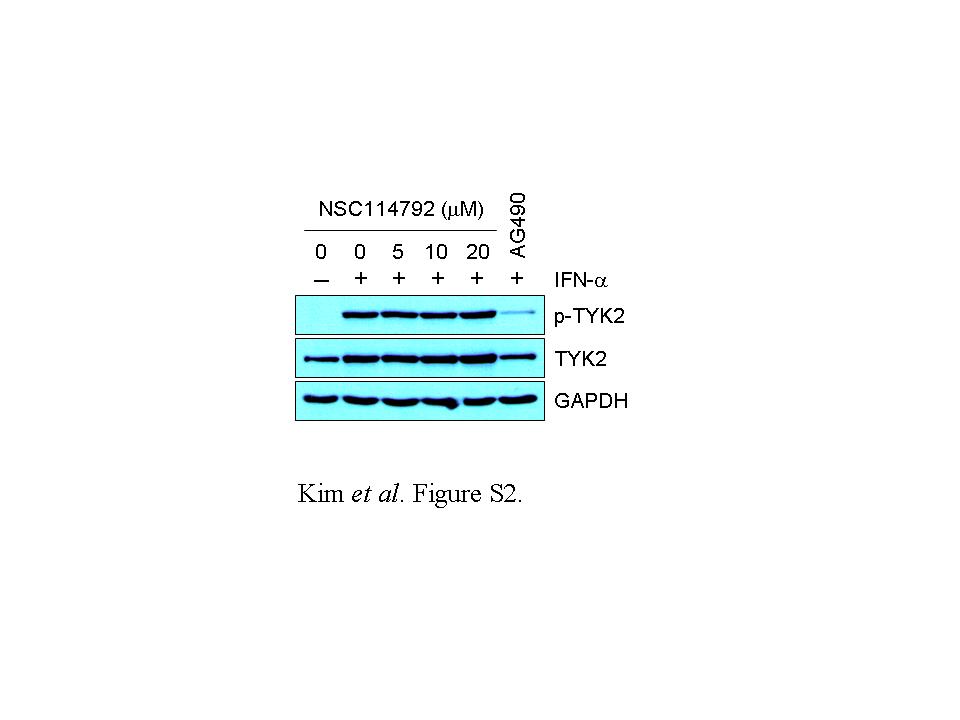

Supplement: Additional file 2 — Figure S2 - Treatment with NSC114792 has no effect on TYK2 phosphorylation. U266 cells were cultured for 24 hours in the presence of either vehicle (DMSO) alone, NSC114792 at different concentrations or the pan-JAK inhibitor AG490 (150 μmol/L), and then stimulated with 1000 U/mL IFN-α for 30 minutes. Whole-cell extracts were processed for Western blot analysis using phospho-TYK2 and TYK2 antibodies. GAPDH serves as a loading control. [file 1476-4598-9-36-S2.JPEG]
